# Synchronization by bleaching does not affect longevity

**DOI:** 10.17912/micropub.biology.001297

**Published:** 2024-09-04

**Authors:** Aura A Tamez González, Abdelrahman AlOkda, Suleima Jacob-Tomas, Jeremy M Van Raamsdonk

**Affiliations:** 1 McGill University, Montreal, Quebec, Canada; 2 Research Institute of the McGill University Health Centre, Montreal, Quebec, Canada

## Abstract

*C. elegans* has been used extensively for research on the biology of aging due to its genetic tractability and short lifespan. In order to measure lifespan, populations of worms are synchronized so that all of the worms being measured begin the assay at the same age. This is typically accomplished by simply picking worms of a particular developmental stage to start the lifespan experiment or through bleaching, a process through which the body of the worm is dissolved in a solution of bleach (sodium hypochlorite) and sodium hydroxide to release its fertilized eggs. In this experiment, we examined the effect of bleaching on lifespan in wild-type worms and the long-lived mitochondrial mutant *isp-1* . We found that bleaching did not affect the longevity of wild-type worms or *isp-1* mutants. While we cannot exclude the possibility that bleaching affects the lifespan of specific genetic mutants, our results indicate that wild-type longevity is unaffected and that for at least some genetic mutants bleaching can be used for synchronization prior to initiating a lifespan experiment.

**Figure 1. Bleaching worms does not affect lifespan.**
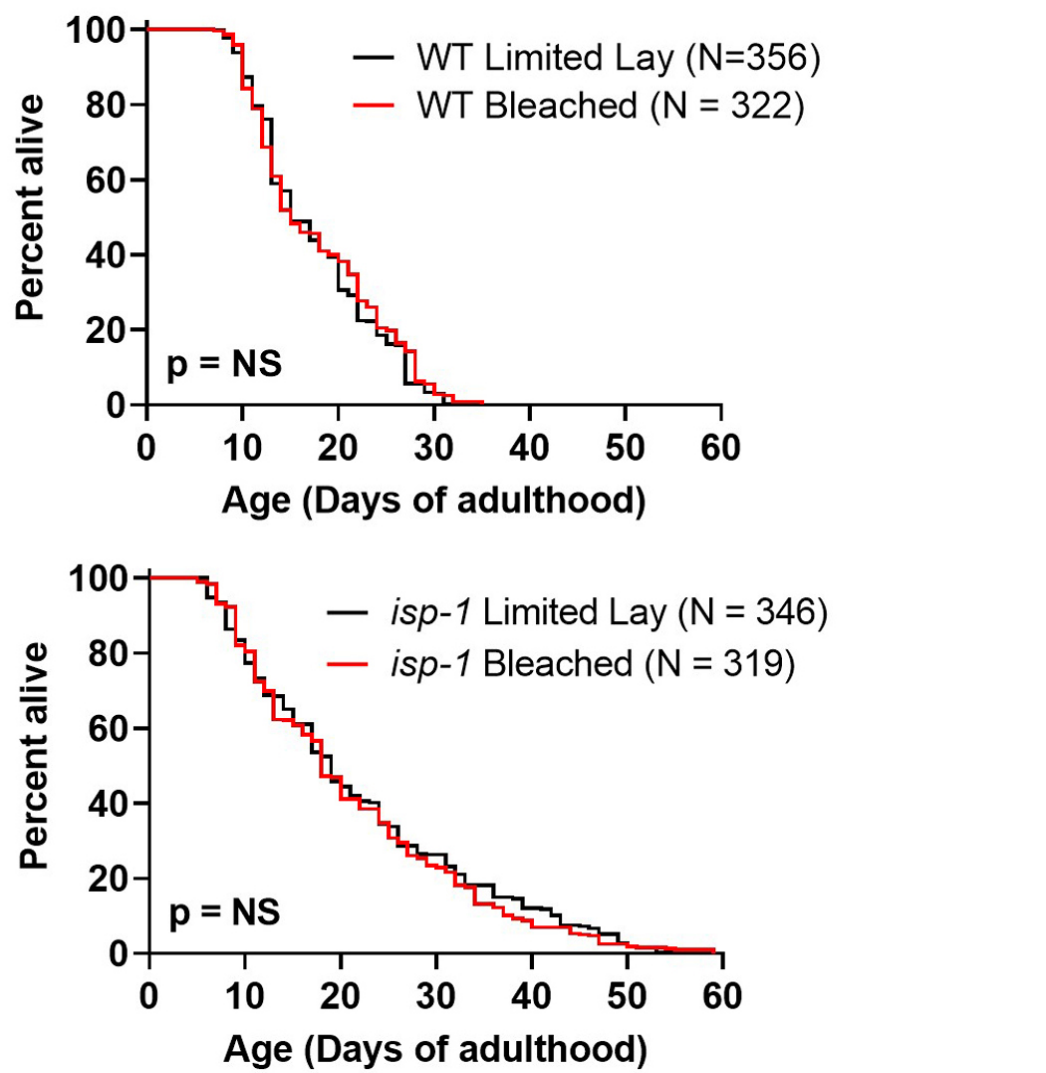
We compared the effect of two different methods of synchronization on worm lifespan using wild-type worms and long-lived *isp-1* mutants *.* For both strains, there was little or no difference in the mean or maximum lifespan between worms that were synchronized using a limited lay compared to worms that were synchronized by bleaching. Four biological replicates were performed by four different experimenters. Statistical significance was assessed using a log rank test. NS = not significant.

## Description

The worm *C. elegans* is a simple genetic model organism that has been used to gain insight into a wide variety of biological processes including aging (Brenner, 1974) . *C. elegans* develop from egg to adulthood through four larval stages L1, L2, L3 and L4. For experiments involving *C. elegans* , it is important to select worms that are at the same developmental stage to ensure the consistency of the results. For experiments measuring lifespan, it is most common to begin the experiment at adulthood in order to separate slow development from extended adult lifespan. To obtain synchronized animals one can either pick worms of the desired developmental stage from a mixed population or limited lay, or one can synchronize the worms by bleaching. Bleaching involves treating gravid adult worms with a solution of sodium hydroxide (NaOH) and bleach (sodium hypochlorite) (Stiernagle, 2006) . The hypochlorite solution dissolves the body of the worm releasing all of the fertilized eggs from inside, as the eggs are protected by their shell. Once the body of the worm has been dissolved the eggs can be rinsed with buffer to remove the hypochlorite solution and allowed to hatch either in liquid or on solid plates. The hatched worms will be synchronized and ready to start the lifespan assay at adulthood.

While both approaches have been used to generate synchronized worms for the lifespan assay, it is not known the extent to which bleaching affects the lifespan of either wild-type or long-lived mutant worms. Accordingly, we sought to determine the effect of bleaching on lifespan, especially since hypochlorite treatment can also cause damage to eggs depending on the exposure (Verdu et al., 2022). Based on this previous work showing a detrimental effect of bleaching on eggs, we hypothesized that bleaching would decrease lifespan. We compared the lifespan of worms picked from an overnight limited lay at the pre-fertile young adult stage to worms that were synchronized by bleaching. We examined wild-type worms and long-lived *isp-1* mutants. *isp-1* worms have a mutation in the gene encoding the Rieske iron sulfur protein, which is part of complex III of the mitochondrial electron transport chain (Feng et al., 2001) . These worms have increased lifespan and stress resistance (Soo et al., 2023) that is at least partially due to the activation of multiple pathways of cellular resilience (Campos et al., 2021; Dues et al., 2017; Harris-Gauthier et al., 2022; Senchuk et al., 2018; Wu et al., 2018) .

We found that the lifespan of wild-type worms is not affected by bleaching ( **Figure 1** ). Similarly, the long-lifespan of *isp-1* mutants is not significantly different when the experimental worms are synchronized by bleaching compared to worms picked from a limited lay ( **Figure 1** ). These results suggest that synchronization by bleaching will not affect the results of a lifespan experiment involving wild-type or *isp-1* worms. It is possible that bleaching may affect the lifespan of specific mutant strains, which would need to be determined experimentally.

## Methods

***C. elegans strains*** . The following strains were used in this study: N2 (wild-type) and *isp-1 ( qm150 ).* Strains were maintained at 20°C on NGM (nematode growth media) plates seeded with OP50 bacteria.

***Bleaching.*** Worms and bacterial lawns with eggs were collected from 6 cm NGM plates for each strain and transferred into a 15 mL centrifuge tube. The tubes were topped up to 15 mL with M9 buffer and centrifuged for 2 minutes at 1300 RCF in a swinging-bucket centrifuge. The supernatant was carefully aspirated, and 2 mL of the bleaching solution (1% bleach, 625 mM NaOH in ddH₂O) was added to the worm pellet. The tubes were vortexed at intervals of 30 seconds at 3000 RPM, not exceeding a total of 6 minutes.

To neutralize and stop the bleaching process, 13 mL of M9 buffer was added to each tube, briefly vortexed, and then centrifuged for 2 minutes at 1300 RCF. The supernatant was aspirated, and the egg pellet was washed by adding 14 mL of M9 buffer, vortexing briefly, and centrifuging for 2 minutes at 1300 RCF. This washing step was repeated three times. After the final wash, the egg pellet was resuspended in 500 µL of M9 buffer and pipetted onto 6 cm NGM plates. Once the progeny reached the young adult stage, they were used in the lifespan experiment.

***Limited Lay.*** For the limited lay, gravid adult worms were placed on an NGM plate overnight (16-24 hours). For the overnight lay, 15 worms were used for wildt-type and 21 worms were used for *isp-1 .* The next day the adults were removed and killed. When the progeny reached the young adult stage they were used in the lifespan experiment.

***Lifespan* .** Lifespan studies were conducted at 20°C on NGM plates containing 25 µM 5-Fluoro-2′-deoxyuridine (FUdR) and seeded with OP50 bacteria. This concentration of FUdR inhibits the development of progeny after transferring to the second FUdR plate and has little or no effect on lifespan (Van Raamsdonk & Hekimi, 2011) . Four replicates were completed by four different experimenters. Each replicate contained two plates with a minimum of 25 worms per plate. Worms were scored as dead if they failed to move after prodding with a platinum wire (worm pick). Worms were first prodded on the tail, then prodded on the head. Worms with internal hatching or externalization of internal organs were censored as were worms that crawled off the plates. Worms were transferred to fresh plates at least twice during the lifespan experiment and were checked every 2-3 days until all of the worms had died. The experimenters were not blind to the treatment or genotype.

***Statistical analysis* .** Statistical analysis was performed using Graphpad PRISM version 9.0 using the log-rank test.

## Reagents

*Strains.*

N2 wild-type (Wild isolate from Bristol)

JVR171 *isp-1 ( qm150* )

*Chemicals* **.**

5-Fluoro-2′-deoxyuridine (F0503, Sigma-Aldrich)

## References

[R1] Brenner S (1974). THE GENETICS OF
*CAENORHABDITIS ELEGANS*. Genetics.

[R2] Campos Juliane C, Wu Ziyun, Rudich Paige D, Soo Sonja K, Mistry Meeta, Ferreira Julio CB, Blackwell T Keith, Van Raamsdonk Jeremy M (2021). Mild mitochondrial impairment enhances innate immunity and longevity through ATFS‐1 and p38 signaling. EMBO reports.

[R3] Dues Dylan J., Schaar Claire E., Johnson Benjamin K., Bowman Megan J., Winn Mary E., Senchuk Megan M., Van Raamsdonk Jeremy M. (2017). Uncoupling of oxidative stress resistance and lifespan in long-lived isp-1 mitochondrial mutants in Caenorhabditis elegans. Free Radical Biology and Medicine.

[R4] Feng Jinliu, Bussière Frédéric, Hekimi Siegfried (2001). Mitochondrial Electron Transport Is a Key Determinant of Life Span in Caenorhabditis elegans. Developmental Cell.

[R5] Harris-Gauthier Namastheé, Traa Annika, AlOkda Abdelrahman, Moldakozhayev Alibek, Anglas Ulrich, Soo Sonja K., Van Raamsdonk Jeremy M. (2022). Mitochondrial thioredoxin system is required for enhanced stress resistance and extended longevity in long-lived mitochondrial mutants. Redox Biology.

[R6] Senchuk Megan M., Dues Dylan J., Schaar Claire E., Johnson Benjamin K., Madaj Zachary B., Bowman Megan J., Winn Mary E., Van Raamsdonk Jeremy M. (2018). Activation of DAF-16/FOXO by reactive oxygen species contributes to longevity in long-lived mitochondrial mutants in Caenorhabditis elegans. PLOS Genetics.

[R7] Soo Sonja K., Traa Annika, Rudich Zenith D., Moldakozhayev Alibek, Mistry Meeta, Van Raamsdonk Jeremy M. (2022). Genetic basis of enhanced stress resistance in long‐lived mutants highlights key role of innate immunity in determining longevity. Aging Cell.

[R8] Stiernagle Theresa (2006). Maintenance of C. elegans. WormBook.

[R9] Van Raamsdonk Jeremy Michael, Hekimi Siegfried (2011). FUdR causes a twofold increase in the lifespan of the mitochondrial mutant gas-1. Mechanisms of Ageing and Development.

[R10] Verdú Samuel, Fuentes Cristina, Barat José M., Grau Raúl (2022). Characterisation of chemical damage on tissue structures by multispectral imaging and machine learning procedures: Alkaline hypochlorite effect in C. elegans. Computers in Biology and Medicine.

[R11] Wu Ziyun, Senchuk Megan M., Dues Dylan J., Johnson Benjamin K., Cooper Jason F., Lew Leira, Machiela Emily, Schaar Claire E., DeJonge Heather, Blackwell T. Keith, Van Raamsdonk Jeremy M. (2018). Mitochondrial unfolded protein response transcription factor ATFS-1 promotes longevity in a long-lived mitochondrial mutant through activation of stress response pathways. BMC Biology.

